# Vitamin D_3_ Regulates the Formation and Degradation of Gap Junctions in Androgen-Responsive Human Prostate Cancer Cells

**DOI:** 10.1371/journal.pone.0106437

**Published:** 2014-09-04

**Authors:** Linda Kelsey, Parul Katoch, Anuttoma Ray, Shalini Mitra, Souvik Chakraborty, Ming-Fong Lin, Parmender P. Mehta

**Affiliations:** Department of Biochemistry and Molecular Biology, University of Nebraska Medical Center, Omaha, Nebraska, United States of America; Emory University School of Medicine, United States of America

## Abstract

1α-25(OH)_2_ vitamin D_3_ (1-25D), an active hormonal form of Vitamin D_3_, is a well-known chemopreventive and pro-differentiating agent. It has been shown to inhibit the growth of several prostate cancer cell lines. Gap junctions, formed of proteins called connexins (Cx), are ensembles of cell-cell channels, which permit the exchange of small growth regulatory molecules between adjoining cells. Cell-cell communication mediated by gap junctional channels is an important homeostatic control mechanism for regulating cell growth and differentiation. We have investigated the effect of 1-25D on the formation and degradation of gap junctions in an androgen-responsive prostate cancer cell line, LNCaP, which expresses retrovirally-introduced Cx32. Connexin32 is expressed by the luminal and well-differentiated cells of normal prostate and prostate tumors. Our results document that 1-25D enhances the expression of Cx32 and its subsequent assembly into gap junctions. Our results further show that 1-25D prevents androgen-regulated degradation of Cx32, post-translationally, independent of androgen receptor (AR)-mediated signaling. Finally, our findings document that formation of gap junctions sensitizes Cx32-expressing LNCaP cells to the growth inhibitory effects of 1-25D and alters their morphology. These findings suggest that the growth-inhibitory effects of 1-25D in LNCaP cells may be related to its ability to modulate the assembly of Cx32 into gap junctions.

## Introduction

The role of Vitamin D_3_, and its active hormonal form 1α-25(OH)_2_ vitamin D_3_ (1-25D), as an anti-neoplastic, pro-differentiating, and pro-apoptotic agent has been established in a wide variety of normal and malignant epithelial cells, including prostate cancer (PCA) [1]–[4]. The actions of 1-25D are mediated by binding to vitamin D receptor, one of the members of nuclear receptor superfamily, which is expressed in a wide variety of cells, including prostate. The vitamin D receptor heterodimerizes with the RXR receptor and binds to vitamin D receptor response element to alter gene expression [1]. Based upon the observation that PCA mortality rates in the U.S are inversely proportional to the geographically incident ultraviolet radiation exposure from the sun, and that ultraviolet light is essential for vitamin D_3_ synthesis in the skin, a role for this vitamin in decreasing the risk of developing PCA has been suggested [5], [6]. Numerous *in vitro* studies show consistent growth inhibitory and differentiation-inducing effects of vitamin D_3_ on prostate carcinoma cells, and animal studies show that it not only reduces the incidence of PCA by acting as a chemopreventive agent but also suppresses metastasis [7]–[10].

Gap junction (GJ)s are ensembles of cell-cell channels that signal non-canonically, by permitting the direct exchange of small molecules (≤1500Da) between the cytoplasmic interiors of contiguous cells [11]. The constituent proteins of GJs, called connexins (Cxs), are coded by 21 genes, which have been designated according to their molecular mass [12]. Cell-cell channels are bicellular structures formed by the collaborative effort of two cells. To form a GJ cell-cell channel, Cxs first oligomerize in the endoplasmic reticulum or the trans-Golgi network as a hexamer, called connexon, which docks with the connexon displayed on a contiguous cell [13], [14]. Multiple lines of evidence now lend credence to the notion that cell-cell communication mediated by gap junctional channels is an important homeostatic control mechanism for regulating cell growth and differentiation and for curbing tumor promotion. For example, impaired Cx expression, or loss of GJ function, has been implicated in the pathogenesis of several types of cancers and diseases [15]–[19]. Also, mutations in several Cx genes have been detected in genetic disorders characterized by aberrant cellular proliferation and differentiation [13], [20].

Our previous studies showed that the expression of Cx32, which is expressed by the luminal cells of the prostate, coincided with the acquisition of the differentiated state of the luminal cells [21], [22]. Moreover, we documented that the progression of PCA from an androgen-dependent state to an invasive, androgen-independent state was characterized by the aberrant trafficking of Cx32 and/or impaired assembly into GJs [22]–[24]. Furthermore, our studies showed that forced expression of Cx32 into androgen-responsive human PCA cell line, LNCaP, retarded cell growth *in vivo* and *in vitro* [22]. We have also shown that in LNCaP cells expressing Cx32, formation and degradation of GJs were regulated by the androgens, which controlled the expression level of Cx32 posttranslationally by preventing its degradation by endoplasmic reticulum associated degradation (ERAD) [25]. Androgens are required to maintain the secretory (differentiation-related) function of the luminal epithelial cells of normal prostate as depletion of androgens by surgical or chemical means triggers apoptosis and/or dedifferentiation of these cells [26]–[29]. Our recent studies have shown that retinoids, which also regulate the proliferation and differentiation of prostate epithelial cells [28], [30], also enhance the assembly of Cx32 into GJs [31]. These studies lend credence to the notion that formation and degradation of GJs may be linked to the proliferation and differentiation of luminal prostate epithelial cells.

Like androgens and retinoids, vitamin D_3_ is essential for the normal development of the prostate and has also been documented to modulate PCA progression [7], [9]. Recent studies have shown that vitamin D suppressed prostatic epithelial neoplasia in Nkx3.1/PTEN transgenic mice [32]. Epidemiologic, cell culture, and clinical studies have implicated antitumor effects of 1-25D for PCA and it has been suggested to be a potent chemopreventive agent [3], [4]. However, in contrast to colon cancer [1], [33], the potential of effectiveness of 1-25D in the chemoprevention of PCA has remained controversial despite numerous studies in transgenic mouse models of PCA and its use in clinical trials [1], [2]. Earlier studies, including ours, have shown that the growth-inhibitory and differentiation-inducing effects of chemopreventive agents might be related to their ability to enhance gap junctional communication [34]–[38]. The luminal cells of normal prostate express Cx32 and form large GJs and progression of PCA is accompanied by loss of ability to form GJs [22], [23]. Formation of GJs has been implicated in maintaining the polarized and differentiated state of epithelial cells [39]. These studies prompted us to examine the effect of 1-25D on the assembly of Cx32 into GJs in androgen-responsive human PCA cell line LNCaP. Because 1-25D has been shown to increase the expression of AR in LNCaP cells [40], we rationalized that it might modulate androgen-regulated formation and degradation of GJs and affect growth of androgen-responsive PCA cells that express Cx32. By using androgen-responsive LNCaP cells, which express retrovirally-introduced Cx32 [25], we show that 1-25D enhances the assembly of Cx32 into GJs. Moreover, we further show that 1-25D prevents androgen-regulated degradation of GJs post-translationally, independent of AR-mediated signaling. Finally, our findings show that formation of GJs sensitizes LNCaP cells to growth-inhibitory effects of 1-25D and alters their morphology.

## Materials and Methods

### Cell Culture

Androgen-responsive human PCA cell line, LNCaP, was grown as described [41], [42]. LNCaP-32 cells, one of the several clones of LNCaP cells expressing retrovirally-transduced rat Cx32, and LNCaP-N cells, one of the several control clones selected in G418 after infection with the control retrovirus, have been described [25], [31]. Parental LNCaP cells, hereafter referred to as LNCaP-P cells, were grown in RPMI containing 5% fetal bovine serum in an atmosphere of 5% CO_2_/95% air whereas LNCaP-N and LNCaP-32 cells were maintained in RPMI containing 5% fetal bovine serum containing G418 at 200 µg/ml as described [25], [31]. Steroid–depleted (charcoal-stripped) serum and phenol-red-free RPMI were obtained from HyClone Laboratories (Salt Lake City, UT).

### Antibodies and Immunostaining

The sources of both monoclonal and polyclonal antibodies against Cx32 have been described previously [24], [25], [31], [43], [44]. Mouse anti-occludin (clone OC-3F10) was from Zymed laboratories, Inc. (South San Francisco, CA). Rabbit antibodies against α- and β-catenin and mouse anti-β-actin (clone C-15) were from Sigma (St. Louis, MO). Monoclonal antibodies against E-cadherin (E-cad), α-catenin, β-catenin, generously provided by Drs. Johnson and Wheelock (Eppley Institute), have been described [25], [43], [44]. A rabbit polyclonal anti-AR receptor antibody was from Santa Cruz Biotech (sc-13062, San Diego, CA). Cells (1.5×10^5^), seeded in six well clusters containing glass cover slips and allowed to grow to approximately 50% confluence, were immunostained with various antibodies as described [24], [25], [31], [43]–[45]. Secondary antibodies (rabbit or mouse), conjugated with Alexa 488 and Alexa 594, were used as appropriate. Images of immunostained cells were acquired with Leica DMRIE microscope (Leica Microsystems, Wetzler, Germany) equipped with Hamamatsu ORCA-ER2 CCD camera (Hamamatsu-City, Japan) and analyzed using image processing software (Volocity, Version 6.3; Improvision, Inc; Perkin Elmer) as described [43]–[45].

### Stock Solutions

Synthetic androgen mibolerone (MB) and a natural androgen dihydro-testosterone (DHT), 1-25D, and Casodex (Bicalutamide) were purchased from BIOMOL (ENZO Life Sciences, Inc., Farmingdale, NY). Stock solutions of MB and DHT were prepared at 1 mM in ethanol and stored at −20°C in small aliquots protected from light. Stock solution of 1-25D (10 µM) was prepared in ethanol and stored in aliquots at −80°C protected from light. Stock solution of Casodex (10 mM) was prepared in DMSO and stored in aliquots at −20°C. They were appropriately diluted in the medium at the time of treatment. All experiments were performed in yellow light as described [36], [37].

### Androgen Depletion and Other Treatments

Cells were seeded in six well clusters with glass cover slips (1.5×10^5^ cells per well) and in 6-cm (2×10^5^ cells per dish) and 10-cm dishes (3.5×10^5^ cells per dish) in 2, 4 and 10 ml complete medium, respectively. Cells were treated by replenishing with fresh medium containing various reagents at the desired concentration when they attained 50% confluence. Controls were treated with ethanol such that the final concentration of the solvent did not exceed 0.1%. When cells were to be grown under androgen-depleted conditions, normal cell culture medium was replaced with androgen-depleted cell culture medium (phenol-red-free RPMI containing 5% charcoal-stripped serum). The controls received fresh phenol-red-free medium containing normal serum. We used phenol-red-free medium because phenol-red has been documented to have steroidogenic effects on the growth of hormone-responsive cell lines, including LNCaP [46], [47].

### Western Blot Analysis and Detergent Solubility of Connexin32

Cells (5×10^5^) were seeded in 10 cm dishes in replicate in 10 ml of complete medium and grown to confluence in the presence and absence of various reagents. Cell lysis, detergent-solubility assay with 1% Triton X-100 (TX100) and the expression level of Cx32 were analyzed by Western blot analysis as described [25], [43], [44]. Briefly, after lysis in buffer SSK (10 mM Tris, 1 mM EGTA, 1 mM PMSF, 10 mM NaF, 10 mM NEM, 10 mM Na_2_VO_4_, 10 mM iodoacetamide, 1% TX100, pH 7.4), total, detergent-soluble and -insoluble extracts were separated by ultracentrifugation at 100,000×g for 60 min (35,000 rpm in analytical Beckman ultracentrifuge; Model 17-65 using a SW50.1 rotor). The detergent-insoluble pellets were dissolved in buffer C (70 mM Tris/HCl, pH 6.8, 8 M urea, 10 mM NEM, 10 mM iodoacetamide, 2.5% SDS, and 0.1 M DTT). Following normalization based on cell number, the total and TX100-soluble and -insoluble fractions were mixed with 4xSDS-loading buffer to a final concentration of 1x and incubated at room temperature for 1 h before SDS-PAGE analysis. Blots were developed with C-Digit (Li-COR, Lincoln, NE) using SuperSignal WestFemto Maximum Sensitivity Substrate (Thermo Scientific; Rockford, IL).

### Communication Assays

Gap junctional communication was assayed by microinjecting Lucifer Yellow (MW 443 Da; Lithium salt), Alexa Fluor 488 (MW 570 Da; A-10436), and Alexa Fluor 594 (MW 760 Da; A-10438) using Eppendorf InjectMan and FemtoJet microinjection systems (models 5271 and 5242, Brinkmann Instrument, Inc. Westbury, NY) mounted on Leica DMIRE2 microscope. After capturing the images of microinjected cells with the aid of CCD camera (Retiga 2000R, FAST 1394) using QCapture (British Columbia, Canada), the permeability of various fluorescent tracers was quantitated by scoring the number of fluorescent cells at 1 min (Lucifer Yellow), 3 min (Alexa 488) and 15 min (Alexa 594) after microinjection into test cell as described [22], [25], [43], [48].

### Colony Formation and Cell Growth Assays

Cell growth was assessed either by colony forming assay or by counting the number of cells as described [22], [48]. For colony forming assay, 1×10^3^ cells were seeded in 6 cm dishes in triplicate in 3 ml culture medium. After 24 h, one ml medium containing 1-25D, MB or DHT was added to the dishes to give the desired final concentration. Cells were grown for 21 days, with a medium change every 4 days containing the appropriate concentration of the above reagents, when they formed visible colonies. Colonies in dishes were fixed with 3.7% buffered formaldehyde, stained with 0.025% solution of crystal violet in PBS, and photographed. For measuring cell growth, 5×10^4^ cells were seeded in 6 cm dishes in replicate and treated with 1-25D described above. Cells were allowed to grow for 10 days with a medium change at day 5. Cells were trypsinized and counted in a hemocytometer.

## Results

### Vitamin D_3_ Enhances Cx32 Expression Level

**Figure 1:**
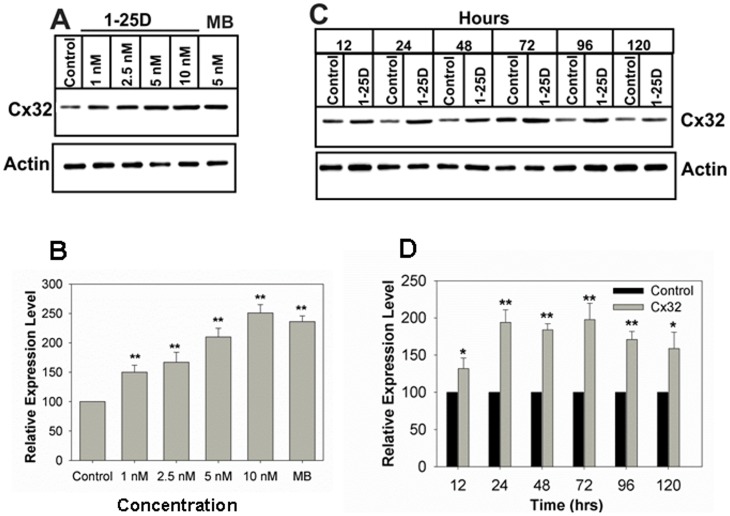
1-25D increases Cx32 expression level. Cx32-expressing LNCaP-32 cells were treated with the 1-25D, 9-CRA, DHT and MB as indicated. **A**. Dose-dependent enhancement of Cx32 expression level upon 1-25D treatment for 48 h. **B**. Quantitative analysis of the expression level of the data shown in **A**. Each bar represents the Mean and the Standard Error of the Mean from 4-17 experiments. Note that significant enhancement is observed even at 1 nM. The asterisks (**) indicate P value of ≤0.0001. A two tailed Student's *t* test was used to calculate P value assuming unequal variance. **C**. Kinetics of enhancement of Cx32 expression level upon treatment with 1-25D (10 nM) for the indicated times. Note that enhancement is observed as early as 12 h and plateaus at 72 h. **D**. Quantitative analysis of the expression level of the data shown in C. Each bar represents the Mean and the Standard Error of the Mean from 3-11 experiments. The asterisk (*) indicates P value of ≤0.0016 and asterisks (**) indicate P value of ≤0.0001. A two tailed Student's *t* test was used to calculate P value assuming unequal variance.

**Figure 2:**
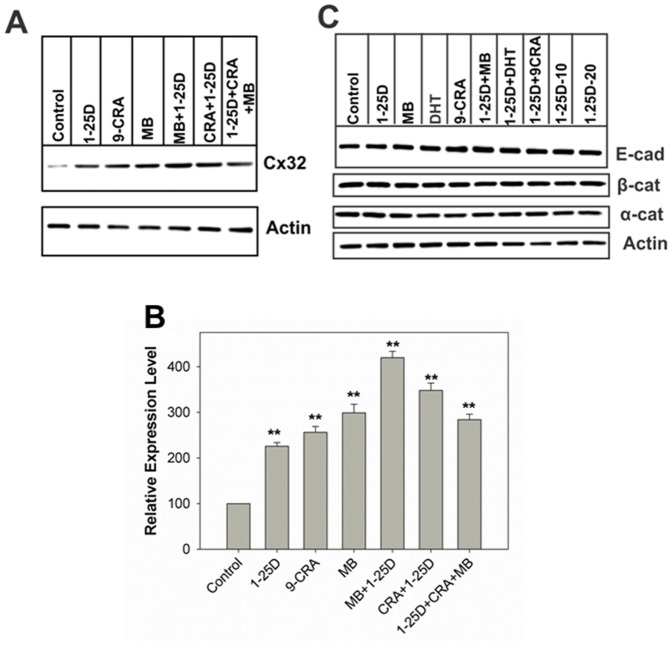
The effect of combined treatment of 1-25D with androgens and retinoids on the expression level of Cx32 and the adherens-junction-associated proteins. Cx32-expressing LNCaP-32 cells were treated with the 1-25D, 9-CRA, DHT and MB as indicated. **A**. Combined treatment with 1-25D with MB or 9-CRA is more effective in increasing Cx32 expression level than treatment with the single agent alone. **B**. Quantitative analysis of the expression level of the data shown in **A**. Each bar represents the Mean and the Standard Error of the Mean from 4-13 experiments. The asterisks (**) indicate P value of ≤0.0001. A two tailed Student's *t* test was used to calculate P value assuming unequal variance. **C**. Effect of 1-25D on adherens junction associated proteins. Expression of adherens junction proteins E-cadherin (E-cad), α-catenin (α-cat), and β-catenin (β-cat) was analyzed by Western blot analysis of total cell lysate (10 µg). Note that there is no effect.

We used LNCaP-32 cells that express retrovirally-transduced rat Cx32 described previously [25], [31]. We previously showed that in LNCaP-32 cells androgens regulated the formation of GJs, post-translationally, by controlling the expression level of Cx32 by inhibiting its ERAD-mediated degradation [25]. Our subsequent studies showed that androgen-regulated degradation of Cx32 was abrogated by all-trans retinoic acid (ATRA) and 9-Cis retinoic acid (9-CRA) [31]. We thus rationalized that 1-25D might act similar to ATRA and 9-CRA. Based on the earlier studies showing the effect of vitamin D on LNCaP cell growth [10], [49]–[52], we treated LNCaP-32 cells with various concentrations of 1-25D to examine its effect on the expression level of Cx32. We found that 1-25D increased Cx32 expression level in a dose-dependent manner (Figure 1A). Significant enhancement was observed even at concentration as low as 1 nM (Figure 1B, left graph). Concentrations higher than 10 nM were toxic to these cells as assessed by the colony formation assay (unpublished data). For subsequent studies we chose 10 nM 1-25D. Time course studies showed that a significant increase in Cx32 expression level occurred as early as 12 h post-treatment with 1-25D and reached a plateau at 72 h (Figure 1CD, right graph). The effect of 1-25D on Cx32 expression level was as potent as of synthetic androgen, MB, and 9-CRA. Moreover, combined treatment with 1-25D and MB was more effective in increasing Cx32 expression level (Figure 2AB). Vitamin D_3_ had previously been shown to affect the expression of level of E-cad, a constituent protein of adherens junctions, in colon cancer cells [33]. To determine if 1-25D also affected the expression level of adherens junction associated proteins, we measured the expression level of E-cad and its associated proteins α- and β-catenins 72 h after treatment with 1-25D. The results showed that 1-25D had no effect on the expression level of E-cad and α- and β-catenins (Figure 2C). As measured by semi-quantitative RT-PCR analysis, 1-25D neither induced the expression of endogenous Cx32 in LNCaP-P or LNCaP-N cells (data not shown) nor altered the expression level of retrovirally-transcribed Cx32 mRNA in LNCaP-32 as documented previously [25].

### Vitamin D_3_ Enhances Gap Junction Assembly and Junctional Communication

**Figure 3:**
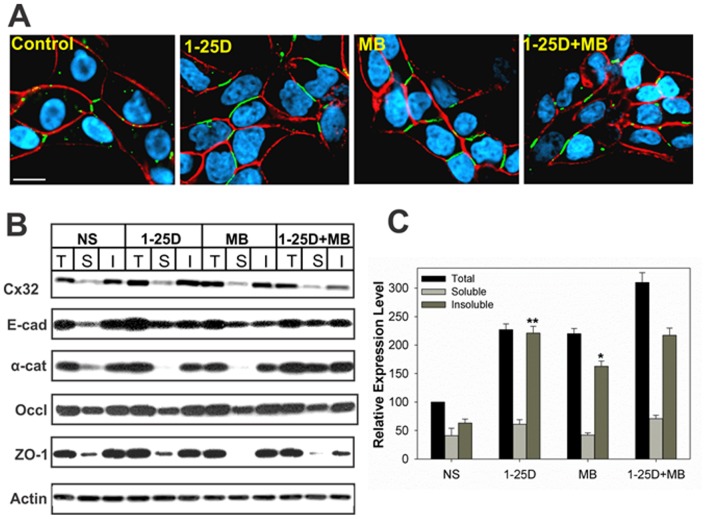
1-25D enhances the assembly of Cx32 into gap junctions. LNCaP-32 cells, grown either in six well clusters or 10-cm dishes, were treated with 1-25D (10 nM), MB (5 nM) and 1-25D plus MB for 48 h. **A**. Assembly of Cx32 (green) into GJs was assessed immunocytochemically. E-cad is shown in red and the nuclei are in blue. Bar = 20 µM. Note that GJ formation was enhanced upon treatment with 1-25D and MB. **B**. TX100- solubility assay was used to measure the assembly of Cx32 into GJs, of tight junction associated protein, occludin (Occl) and ZO-1, and the adherens junction protein, E-cadherin (E-cad) and α-catenin (α-cat). T = total fraction; S = soluble fraction and I = Insoluble fraction. **C**. Quantitative analysis of the expression level of Cx32 shown in **B**. Each bar represents the Mean and the Standard Error of the Mean from 4-17 experiments. Note that both the total level and the detergent-insoluble fraction of Cx32 increased significantly. Each bar represents the Mean and the Standard Error of the Mean from 3-11 experiments. The asterisk (*) indicates P value of ≤0.0016 and asterisks (**) indicate P value of ≤0.0001. A two tailed Student's *t* test was used to calculate P value assuming unequal variance. Note the absence of effect on adherens junction associated proteins, E-cad, and tight junction associated protein, occludin (Occl).

We next examined the effect of 1-25D on the assembly of Cx32 into GJs. We found that, concomitant with an increase in the expression level of Cx32, 1-25D also increased GJ assembly as assessed by immunocytochemical analysis (Figure 3A) and biochemically by Western blot analysis of total, TX100-soluble and –insoluble extracts at 48 h after treatment (Figure 3BC). This biochemical method is based on the principle that Cxs, which are incorporated into GJs, become insoluble in TX100 whereas Cxs that are not incorporated into GJs remain soluble [53]. This assay has been reproducibly shown to measure the assembly of Cxs into GJs as documented by earlier studies [24], [25], [53]. Moreover, we found that enhancement of GJ assembly was accompanied by a 2-3 fold parallel increase in junctional communication as determined by the junctional transfer of three GJ permeable fluorescent tracers, Lucifer Yellow (MW 443), Alexa 488 (MW 570), and Alexa 594 (MW 760). For example, 1-25D increased junctional transfer of Alexa 594 by 2-3 folds compared to controls (Table 1). The effect of 1-25D on junctional communication was as potent as of synthetic androgen, MB, and the natural androgen DHT (Table 1). To determine if 1-25D affected the assembly of other junctional complexes, we also examined the detergent-solubility of adherens and tight junction associated proteins. The rationale behind these studies was that E-cad has been shown to facilitate the assembly of Cxs into GJs [43], [44], [54], and Cx expression has been shown to facilitate the assembly of tight junctions and their constituent proteins [39]. We found that 1-25D had no effect on the solubility of E-cad and its associated proteins, α- and β-catenin, and tight junction associated proteins, ZO-1 and occludin, in TX-100 suggesting that their assembly was not further enhanced into respective cell junctions (Figure 3B). Taken together, these data suggest that 1-25D, like androgens and retinoids, enhances the expression level of Cx32, and its subsequent assembly into GJs, without discernibly altering the expression level of other cell junction associated proteins.

**Table 1:** Effect of 1,25D and androgen on the junctional transfer of fluorescent tracers in LNCaP-32 cells.

Junctional Tracer	Expt #	Junctional Transfer a			
		NS	NS+1-25D^b^	NS+DHT b	NS+MB b
Lucifer Yellow (MW 443)	1 2	11.3±2.1(17)^c^ 13.1±3.1(19)^c^	27.4±3.1(19)^c^ 29.7±5.3(18)^c^	25.6±5.1(21)^c^ 29.1±7.3(27)^c^	34.7±5.7(22)^c^ 38.1±8.3(20)^c^
Alexa-488 (MW 570)	1 2	14.1±5.3(17)^c^ 11.3 ±3.9 (22)c	26.7±4.9(27)^c^ 21.3±4.2(24)^c^	26.9.±6.9(23)^c^ 23.5±5.1(28)^c^	31.2±7.1(24)^c^ 27.3±8.2(29)^c^
Alexa-594 (MW 760)	1 2	6.2±2.1 (20)^c^ 7.7±2.7(27)^c^	19.3±3.3(22)^c^ 16.5±4.5(29)^c^	15.4±3.8(27)^c^ 17.7±5.4(19)^c^	13.7±2.3(23)^c^ 14.9±4.1 (26)^c^

LNCaP-32 cells, seeded in 6 cm dishes in replicate, were grown to 65–70% confluence. Junctional transfer was measured after microinjecting fluorescent tracers (see Materials and Methods).

a The number of fluorescent cell neighbors (Mean ± SE) 1 min (Lucifer Yellow), 3 min (Alexa-488) and 15 min (Alexa-594) after microinjection into test cell. The total number of injection trials is shown in parentheses.

b Cells were treated for 48 h with 1-25D, DHT (10 nM) and MB (2.5 nM).

c P≤0.0001 for normal serum versus stripped and treated cells. A two tailed Student's *t* test was used to calculate P value assuming unequal variance.

### Vitamin D_3_ Modulates Androgen-regulated Formation and Degradation of Gap Junctions

**Figure 4:**
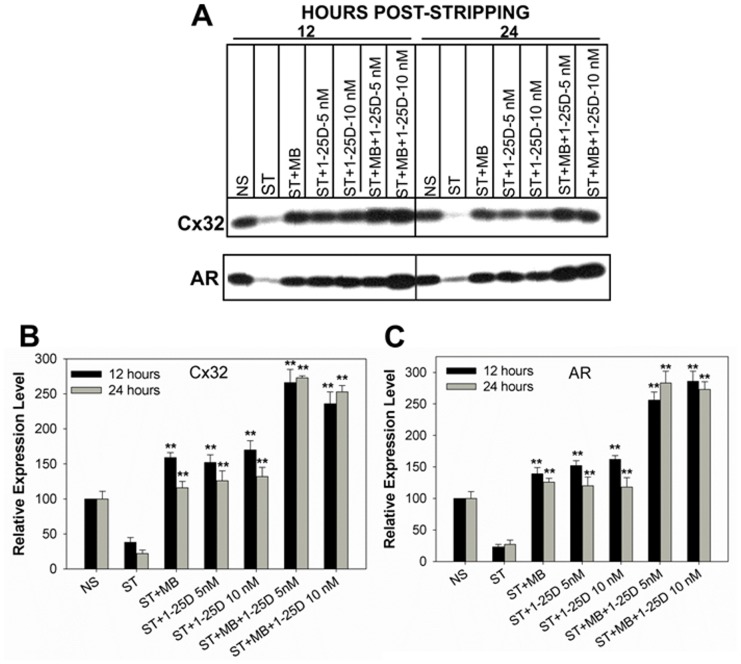
1-25D blocks androgen-regulated degradation of Cx32. LNCaP-32 cells, seeded in 10 cm dishes, were switched to charcoal-stripped (androgen-depleted) medium (ST). Expression level of Cx32 and AR were determined by Western blot analysis (**A**) in the presence and absence of 1-25D (10 nM) and MB (5 nM). Note that Cx32 and AR are degraded upon androgen depletion and degradation is blocked upon 1-25D treatment. **BC**. Quantitative analyses of the expression level of Cx32 (**B**) and AR (**C**) of the data shown in **A**. Each bar represents the Mean and the Standard Error of the Mean from 5-11 experiments. The asterisks (**) indicate P value of ≤0.0001. A two tailed Student's *t* test was used to calculate P value assuming unequal variance.

**Figure 5:**
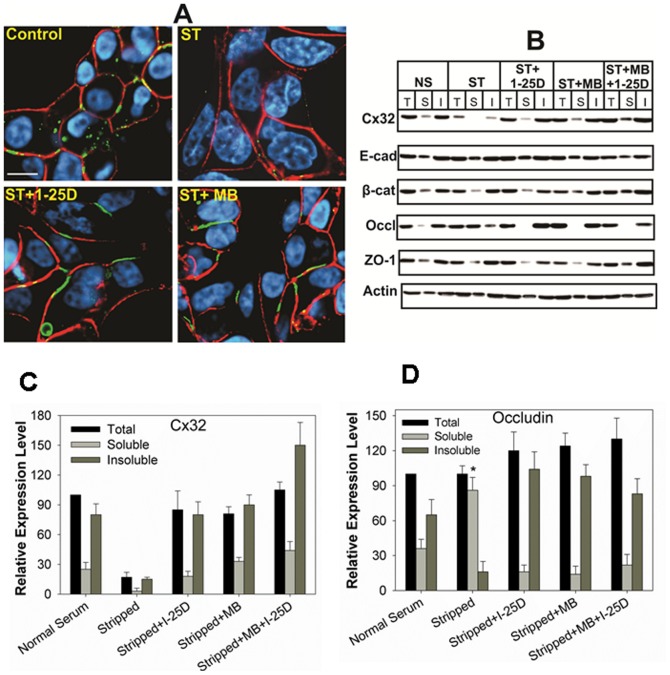
1-25D blocks androgen-regulated degradation of Cx2 and gap junctions. LNCaP-32 cells, seeded in six well clusters or 10 cm dishes, were switched to charcoal-stripped, androgen-depleted medium (ST). GJ assembly and the expression level of Cx32 were determined by immunocytochemical (**A**) and Western blot (**B**) analyses by TX100-solubility assay in the presence and absence of 1-25D (10 nM) and MB (2.5 nM). In (A), Cx32 is in green and E-cad is red and the nuclei (blue) are stained with DAPI. Scale bar in A = 20 µM. In (B), T = total fraction; S = soluble fraction and I = Insoluble fraction. TX100-soluble and insoluble fractions as well as immunocytochemical assay were performed 24 h post-stripping as described in Materials and Methods. Note that GJs are degraded upon androgen depletion and degradation is blocked upon 1-25D treatment (**A**). Note also that the TX100-soluble fraction of E-cad (E-cad), β-catenin (β-cat) and ZO-1 is not affected. Note also that androgen-depletion increases the soluble fraction of occludin (**B**) without affecting the total occludin levels as quantitated in **D**. The asterisk (*) indicates P value of ≤0.0016 and asterisks (**) indicate P value of ≤0.0001. A two tailed Student's *t* test was used to calculate P value assuming unequal variance.

Earlier studies with LNCaP-32 cells had shown that androgen depletion caused degradation of Cx32 by ERAD, and that androgens enhanced GJ formation by re-routing the ERAD-targeted pool of Cx32 to the cell surface, making it amenable for GJ assembly [25]. In subsequent studies, we showed that androgen-regulated formation and degradation of GJs was prevented by 9-CRA and ATRAs [31]. We rationalized that 1-25D might enhance GJ assembly by rescuing the ERAD-targeted pool of Cx32 like 9-CRA and ATRA. Therefore, we examined the expression level of Cx32 and its assembly into GJs upon androgen depletion in the presence and absence of 1-25D in LNCaP-32 cells. For these studies, we used androgen-depleted (charcoal-stripped) and phenol-red-free cell culture medium to grow cells because phenol-red has a weak steroidogenic effect [46]. As was observed in our earlier studies [25], we found that androgen-depletion decreased the expression level of Cx32 within 12 h, which not only was prevented upon addition of MB but also by 1-25D (Figure 4AB). Moreover, combined treatment with MB and 1-25D appeared to be more effective in enhancing the expression level of Cx32 (Figure 4A, upper blot). We also found that androgen depletion decreased the expression level of AR, which was also prevented upon treatment with not only androgens but also with 1-25D (Figure 4A, bottom blot). To substantiate the above data, we further assessed the formation of GJs immunocytochemically (Figure 5A) and functionally by measuring the junctional transfer of Lucifer Yellow, Alexa 488, and Alexa 594 (Table 2). The results showed that GJs were barely observed in cells grown in androgen-depleted medium as assessed by the lack of Cx32-specific immunostaining at cell-cell contact areas, while they were readily observed when androgen-depleted medium was supplemented with 1-25D and MB (Figure 5A). Functional assays showed that the junctional transfer of Lucifer Yellow, Alexa 488 and Alexa 594 decreased significantly upon androgen depletion, which was prevented upon replenishing androgen-depleted medium with MB and 1-25D (Table 2), thus substantiating the immunocytochemical data.

**Table 2:** Effect of 1-25D and androgens on junctional transfer of fluorescent tracers in LNCaP-32 cells under androgen-depleted conditions.

Treatment	Exp #	Junctional Transfer^a^		
		LY (MW 443)	Alexa 488 (MW 570)	Alexa-594 (MW 760)
NS	1 2	13.3±3.7(31) 12.2±2.5(32)	23.7±4.3(29) 29.1±6.4(23)	17.7±3.9(27) 15.3±5.4(21)
Strip^b^	1 2	1.9±0.6(25)c 2.5±0.9(28)c	2.9±0.3(26)c 2.3±0.7.(22)c	1.1±0.3(21)c 00± 0(17)c
Strip+MB	1 2	27.3±.4.5(37)d 31.7±5.4(26)d	27±3.9(28)d 34.1±6.7(24)d	16.1±2.9(27)d 17.1±3.9(29)d
Strip+1-25D	1 2	29.7±4.9(20)d 30.1±6.1(26)d	33.7±7.6(22)d 30.2±5.5(29)d	14.7±4.1(23)d 15.1±5.3 (22)d
Strip+DHT	1 2	19.2±2.9(26)d 23.1±4.3(20)d	27.2±6.3(27)d 29.8±6.1(23)d	18.1±5.1(33)d 15.6±5.3(26)d

LNCaP-32 cells were seeded as described in Table 1 legend. Cells were switched to charcoal-stripped, androgen-depleted medium (Strip) for 48 h in the presence and absence of 1-25D and synthetic (MB) and the natural (DHT) androgens.

a The number of fluorescent cell neighbors (mean ± SE) 1 min (Lucifer Yellow), 3 min (Alexa-488) and 15 min (Alexa-594) after microinjection into test cell. The total number of injection trials is shown in parentheses.

b Cells were stripped for 24 h in the presence and absence of 1-25D, DHT (10 nM) and MB (2.5 nM).

c P≤0.0001 for normal serum (NS) versus stripped (Strip) for all tracers. A two tailed Student's *t* test was used to calculate P value assuming unequal variance.

d P≤0.0001 for stripped serum (Strip) versus stripped and treated cells for all the tracers. A two tailed Student's *t* test was used to calculate P value assuming unequal variance.

The immunocytochemical and junctional transfer data were further corroborated by the TX100-solubility assay (Figure 5BC). We also examined the effect of androgen-depletion on the detergent-solubility of E-cad and β-catenin and tight-junction-associated proteins, ZO-1 and occludin. Consistent with our earlier studies [25], the results showed that androgen depletion increased the detergent-solubility of occludin but not of ZO-1 and E-cad and β-catenin (Figure 5BD). We also examined the effect of MB and 1-25D either alone or in combination on the formation of GJs in parental LNCaP-P and G418-resistant LNCaP-N cells and found that they had no effect (data not shown). Collectively, these data suggest that 1-25D prevents androgen-regulated degradation of Cx32 and enhances GJ formation in LNCaP-32 cells. Because combined treatment with androgens and 1-25D did not enhance GJ assembly further, it is likely that the assembly was enhanced by rescuing the same pool of Cx32 that was targeted for ERAD upon androgen depletion. Moreover, as was observed in our earlier studies, the assembly and detergent-solubility of Cx32 and occludin into cell junctions, or vice versa, appears to be regulated coordinately [25].

### 1-25D Enhances Gap Junction Formation Independent of Androgen Receptor Function

**Figure 6:**
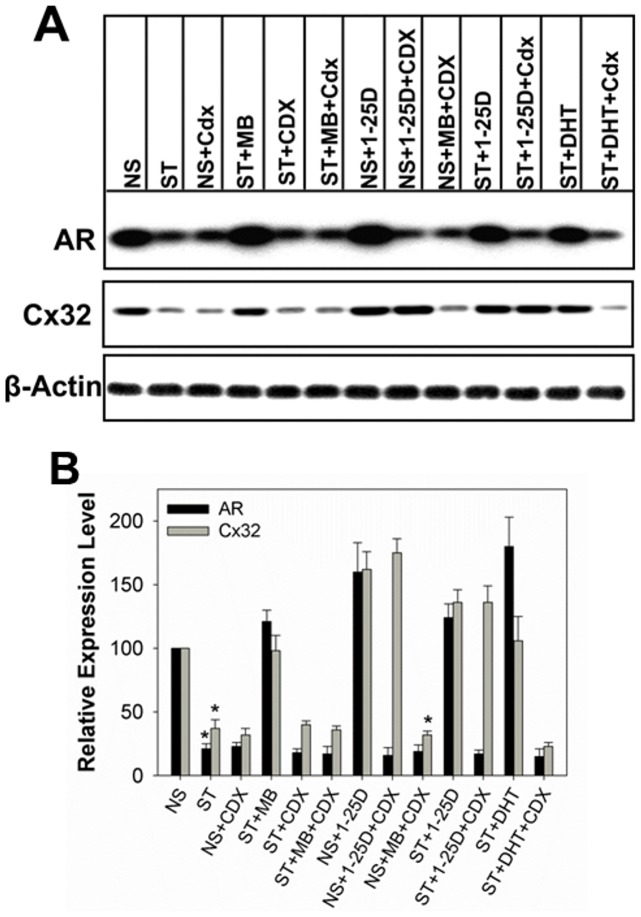
Effect of 1-25D and MB on the expression level of Cx32 and AR in the presence and the absence of Casodex. LNCaP-32 cells, seeded in 6-cm dishes, were grown to 70% confluence. Cells were then grown for additional 24 h in normal medium (NS), androgen-depleted medium alone (ST), normal serum supplemented with Casodex (CDX; NS+CDX), androgen-depleted medium supplemented with MB (ST+MB), MB and Casodex (ST+MB+CDX), 1-25D (ST+1-25D), 1-25D and Casodex (ST+1-25D+CDX) and in normal serum with 1-25D (NS+1-25D). Expression level Cx32 and AR were analyzed by Western blotting (**A**). Note that Cx32 is not degraded in cells treated with 1-25D both in the presence and absence of Casodex whereas it is degraded in normal serum and androgen-depleted but MB supplemented medium containing Casodex. **B**. Quantitative analyses of the expression level of Cx32 and AR of the data shown in **A**. Each bar represents the Mean and the Standard Error of the Mean from 3-9 experiments. The asterisks (*) indicate P value of ≤0.0001. A two tailed Student's *t* test was used to calculate P value assuming unequal variance.

**Figure 7:**
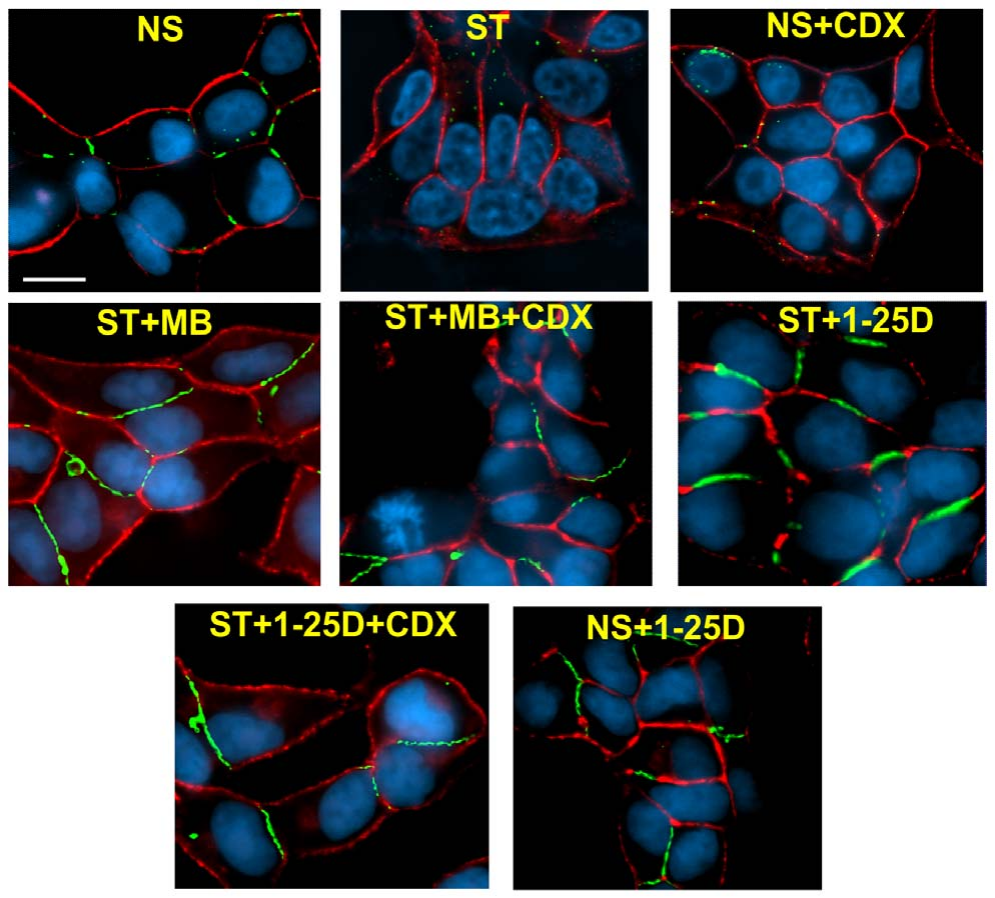
Effect of 1-25D and MB on the formation of gap junctions in the presence and the absence of Casodex. LNCaP-32 cells, seeded on glass cover slips, were grown to 70% confluence. Cells were then grown for additional 24 h in normal medium (NS), androgen-depleted medium alone (ST), normal serum supplemented with Casodex (CDX; NS+CDX), androgen-depleted medium supplemented with MB (ST+MB), MB and Casodex (ST+MB+CDX), 1-25D (ST+1-25D), 1-25D and Casodex (ST+1-25D+CDX) and in normal serum with 1-25D (NS+1-25D). Gap junction formation was assessed immunocytochemically. Note that GJs (green) are not degraded in cells treated with 1-25D both in the presence and absence of Casodex whereas they are degraded in normal serum and androgen-depleted but MB supplemented medium containing Casodex. E-cad is shown in red and the nuclei (blue) are stained with DAPI. Scale Bar = 20 µM.

Our data showed that 1-25D prevented the degradation of AR upon androgen depletion (Figure 4A, bottom blot). Also treatment of LNCaP cells with 1-25D had been shown to enhance AR expression level [40]. Thus, we considered the possibility that the effect of 1-25D on the enhancement of GJ assembly depended on the function of AR alone — and not on the independent effect of 1-25D. To test this notion, we treated LNCaP-32 cells with the anti-androgen, Casodex (Bicalutamide), to block androgen action [55], [56]. Both androgen depletion and treatment with Casodex caused degradation of AR (Figure 6A, upper blot, Figure 6B) and abolished the effect of MB and DHT on Cx32 expression level (Figure 6A, bottom blot; Figure 6B) as was observed in our earlier studies [25], [31]. However, we found that Casodex had no effect on the enhancement of the expression level of Cx32 resulting from the treatment with 1-25D in androgen-depleted medium (Figure 6AB). To substantiate these data, we next examined the formation of GJs immunocytochemically in cells treated with Casodex in the presence and absence of MB or 1-25D. We found that GJs were not formed when cells were treated with Casodex in normal serum or in androgen-depleted medium containing MB as was observed in our earlier studies (Figure 7) [25], [31]. On the other hand, we found that GJs were abundantly formed when cells were treated with Casodex and 1-25D (Figure 7). Altogether, these data suggest that the mechanism by which 1-25D prevents the degradation of Cx32 and enhances GJ formation upon androgen depletion is independent of AR.

### Connexin32 Expression Alters the Growth Response of LNCaP Cells to 1-25D

**Figure 8:**
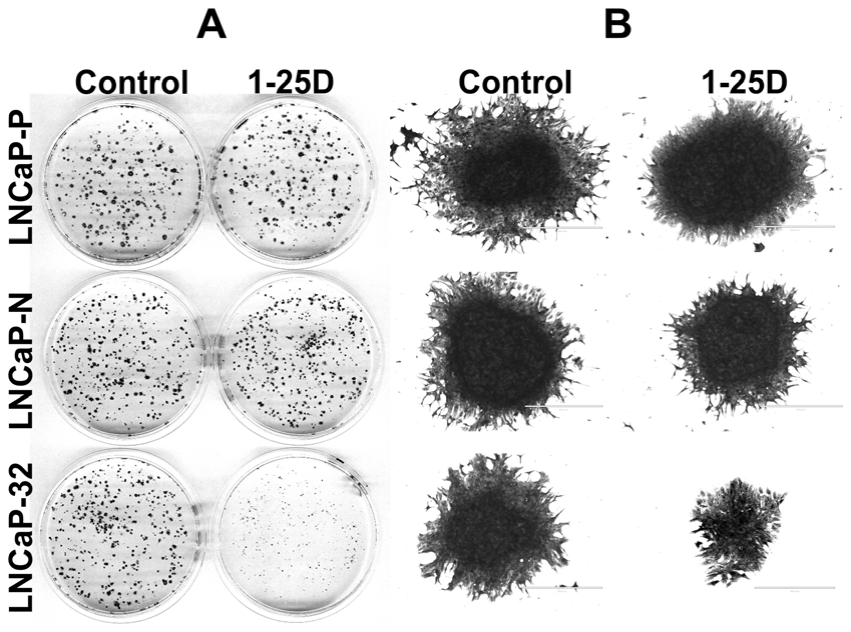
Connexin32 expression and junction formation augments the growth inhibitory effect of 1-25D. LNCaP-P, LNCAP-N and LNCaP-32 cells were seeded at clonal density (2×10^3^) and treated with 1-25D (1 nM) after 24 h. Cells were grown for 21 days with a medium change every 4 days when they formed colonies. Colonies were fixed and stained with crystal violet. **A**. Representative dishes showing colonies. **B**. Morphology of the individual colonies at higher magnification. Note the robust decrease in colony size in 1-25D-treated dishes.

**Figure 9:**
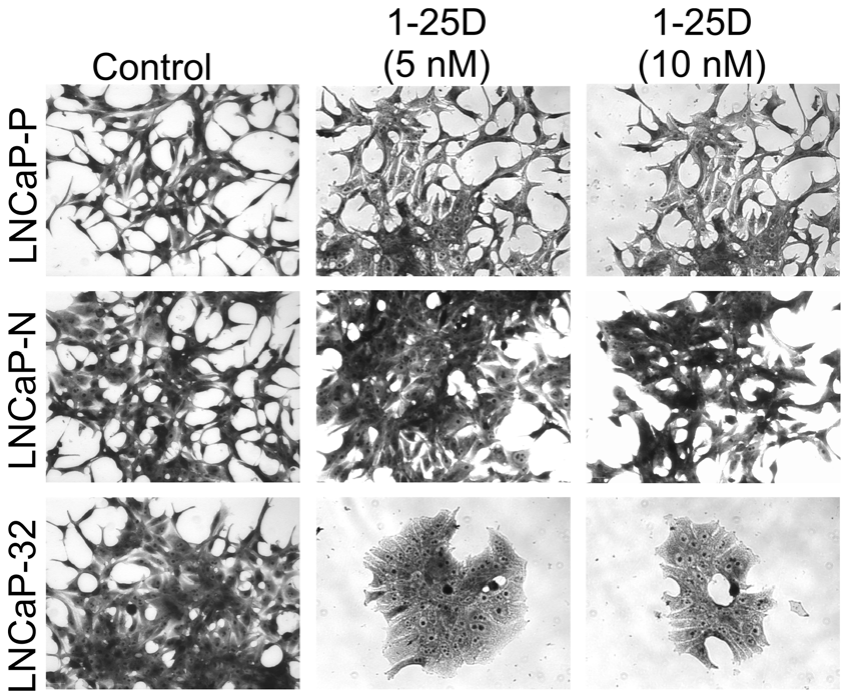
1-25D alters the morphological phenotype of Cx32-expressing LNCaP cells. LNCaP-P, LNCaP-N and LNCaP-32 cells were seeded at a density of 2-3×10^4^ per 6-cm dish and treated with the indicated concentrations of 1-25D after 24 hrs. After 5 days, cells were fixed and stained with crystal violet. Note robust morphological changes in LNCaP-32 cells treated with 1-25D.

Our earlier studies showed that Cx32 expression potentiated the growth-inhibitory effect of 9-CRA and ATRA in LNCaP cells [31]. To test if 1-25D has similar effect on growth, we measured cell growth of LNCaP-32 cells at concentrations that were barely growth-inhibitory to LNCaP-P cells. We determined cell growth by the colony forming assay as well as by counting the number of cells (Figure 8, Table 3). As assessed visually by the size of the colonies, we found that the growth of LNCaP-32 cells was inhibited by 1-25D whereas the growth of LNCaP-P and LNCaP-N cells was minimally affected (Figure 8AB). These data were substantiated by measuring the growth of LNCaP-32 cells at two different concentrations (Table 3). For example, the growth of LNCaP-P and LNCaP-N cells was inhibited by only 20–25% upon treatment with 1-25D (1nM and 2.5 nM) whereas the growth of LNCaP-32 cells was inhibited by 55–70%. Moreover, we found that higher concentrations of 1-25D (5 and 10 nM) altered the morphology of LNCaP-32 cells profoundly such that LNCaP-32 cells treated with 1-25D appeared flatter and more epithelial-like whereas these changes were minimally observed in LNCaP-P and LNCaP-N cells (Figure 9). Change occurred only in response to 1-25D; and only when cells had been growing in 1-25D-containing medium for at least 4 days and have begun to be contact-inhibited and growth-arrested.

**Table 3:** Cx32 expression sensitizes LNCaP cells to growth inhibitory effect of 1-25D.

Treatment	LNCaP-P	LNCaP-N	LNCaP-32
Experiment # 1			
Control	7.9±1.5 (100±19)a	7.1±1.1 (100±15)a	6.5±1.6 (100±25)b
MB (2.5 nM)	6.1±1.4 (77±23)a	6.2±1.2 (87±19)a	3.7±0.8 (57±22)b
1-25D (1 nM)	6.8±1.3 (86±19)a	6.8±1.1 (96±16)a	2.9±0.3 (45±10)b
1-25D (2.5 nM)	5.9±0.9 (75±15)a	6.3±1.0 (89±16)a	1.9±0.4 (29±21)b
Experiment #2			
Control	7.7±1.8 (100±23)a	7.1±1.7 (100±24)a	6.7±1.5 (100±22)b
MB (2.5 nM)	6.7±1.1 (87±16)a	6.3±1.2 (89±19)a	3.8±0.6 (57±16)b
1-25D (1 nM)	6.2±0.6 (81±10)a	6.6±1.3 (93±20)a	2.8±0.8 (42±29)b
1-25D (2.5 nM)	6.6±0.9 (86±14)a	6.2±1.5 (87±24)a	2.2±0.7 (33±32)b
Experiment # 3			
Control	8.3±1.5 (100±18)a	7.4±1.9 (100±26)a	6.9±1.0 (100±14)b
MB (2.5 nM)	7.2±1.0 (87±14)a	6.6±1.4 (89±21)a	3.2±0.7 (46±22)b
1-25D (1 nM)	6.8±1.3 (82±16)a	6.8±1.1 (92±16)a	2.6±0.9 (38±34)b
1-25D (2.5 nM)	6.9±1.1 (86±16)a	6.4±1.2 (86±19)a	2.3±0.5 (33±22)b

LNCaP-P, LNCaP-N and LNCaP-32 cells were seeded in 6-cm dishes in replicate (5×10^4^ cells/dish) and treated with 1-25D (1 nM or 2.5 nM) and MB (2.5 nM). Cells were grown for 10 days with a medium change at day 2 and 5. Cells were trypsinized and counted as described in Materials and Methods. The values represent Mean number of cells per dish ×10^5^ ± SE of the Mean. Values in the parentheses represent Means of % Growth ± SE of the Mean.

a P≥0.12.

b P≤0.05.

## Discussion

The main findings of this study are as follows: 1. 1-25D enhances the expression level of Cx32 and its assembly into functional GJs in androgen-responsive LNCaP cells through inhibition of Cx32's degradation. 2. Formation of GJs sensitizes LNCaP cells to the growth inhibitory effect of 1-25D. We previously showed that 9-CRA and ATRA, the two well-known chemopreventive agents, also enhanced GJ assembly and sensitized these cells to their growth inhibitory effects [31]. Thus, it appears that GJ assembly is also the down-stream target of 1-25D in androgen-responsive LNCaP-32 cells, leading to suppression of growth. Several independent lines of inquiry prompted us to undertake these studies. First, growth inhibitory and chemopreventive effects of retinoids and vitamin D_3_ had been previously documented to correlate with their ability to enhance the assembly of Cxs into GJs in other cancer cell types [35]–[37], [57]. Second, the differentiated and polarized state of epithelial cells of the prostate, as well as of several other exocrine glands and tissues, had generally been found to coincide with the expression of Cx32 and its assembly into GJs [22], [23], [58]. Third, numerous studies had shown that like androgens [28], [29], retinoids [57], [59]–[63], and 1-25D were essential for the growth and differentiation of the prostate [4], [7], [64]. Hence, we rationalized that its expression and assembly into GJs might as well as be regulated by 1-25D either alone or in conjunction with the androgens.

How might 1-25D enhance GJ assembly in LNCaP-32 cells? Nearly 50% of newly synthesized Cx32 may be degraded in the endoplasmic reticulum by ERAD [65]. We had previously shown that in LNCaP-32 cells, androgen depletion caused the degradation of nearly 70–80% of Cx32 by ERAD, and degradation was prevented upon replenishment with the androgens, which allowed Cx32 to traffic to the cell surface and assemble into GJs [25]. These studies further showed that androgens neither induced the expression of Cx32 in Cx-null LNCaP-P cells nor increased the expression level of retrovirally-driven Cx32 in LNCaP-32 cells [25], [31]. Thus, in LNCaP-32 cells, androgens enhanced the expression level of Cx32 posttranslationally [25]. Although not tested directly, our data seem to suggest that 1-25D also enhances GJ assembly by preventing the androgen-regulated degradation of Cx32 by ERAD posttranslationally both under normal and androgen-depleted conditions. Like androgens, 1-25D neither induced the expression of the endogenous Cx32 in LNCaP-32 cells nor affected retroviral driven Cx32 mRNA transcripts. Our previous studies with LNCaP-32 cells showed that AR-mediated signaling was the sole determining factor in enhancing Cx32 expression level and preventing GJ degradation both under androgen-depleted or androgen-containing medium as Casodex, which inhibits AR-function [66], annulled the effect of androgens on Cx32 expression level and its subsequent assembly into GJs [25]. With regard to 1-25D effect, our data showed that it enhanced the expression level of AR under androgen-depleted conditions (Figure 4). Therefore, it is possible that the effect of 1-25D in the absence of androgens may be indirectly caused by persistent and increased level of AR and its activation by the trace amounts of androgens present in the charcoal-stripped medium. However, 1-25D also enhanced GJ assembly robustly in the presence Casodex in androgen-depleted medium, which robustly decreased AR level and inhibited AR function (Figure 6). One plausible explanation for these findings is that 1-25D activates an AR-dependent mechanism under androgen-depleted conditions to rescue the ERAD-targeted pool of Cx32 yet triggers another signaling pathway to enhance GJ assembly when AR function is inhibited by Casodex both under normal and androgen-depleted conditions. Further studies are required to explore this possibility. Of note here are the findings that similar effects were also observed with 9-CRA and ATRA [31].

Cadherins have been shown to facilitate the assembly of Cxs into GJs. However, we failed to observe any effect of 1-25D on the expression and degradation of adherens junction associated proteins, E-cad and its associated proteins α and β catenin under androgen-containing and androgen-depleted conditions (Figures 3 and 5). Therefore, E-cad and its assembly into adherens junctions are not the likely targets of 1-25D in enhancing GJ assembly in contrast to its effect on E-cad expression in human colon carcinoma cells [33]. The assembly of Cx32 into GJs has also been shown to affect tight junction assembly [39], [67]. Our previous studies had shown that androgen depletion increased the detergent-solubility of occludin, without significantly altering its expression level, and that the trafficking of occludin to the cell surface and its detergent-solubility was controlled by the assembly of Cx32 into GJs under androgen-depleted conditions [25]. Similar observations were also made in other cell lines [39], [68], [69]. The present study also showed that androgen-depletion increased the detergent-soluble fraction of occludin without altering its expression level, which was negated when LNCaP-32 cells were treated with 1-25D (Figure 5BD). In this regard, our data suggest that the assembly of Cx32 and occludin might as well be coordinately regulated by 1-25D, and that this may be one of the additional mechanisms by which 1-25D maintains the polarized state of prostate epithelial cells and acts as a differentiating and chemopreventive agent. Further studies are required to substantiate this notion.

1-25D has been shown to induce G0/G1 arrest, differentiation and apoptosis of tumor cells by modulating different signaling pathways to delay tumor progression in different cancer cell types; moreover, it has also been known to potentiate the cytotoxic effects of many chemotherapeutic agents [1]–[3]. Several studies have shown that vitamin D inhibits the growth of PCA cell lines, including LNCaP, in both AR-dependent and –independent manner [10], [49], [50], [70], [71]. We found that the expression of Cx32 potentiated the growth inhibitory effect of 1-25D such that suppression of growth was observed at doses which had no significant effect on the growth of Cx-null LNCaP cells (Figure 8, Table 3). For example, 1-25D at 1nM barely inhibited the growth of LNCaP-P and LNCaP-N cells but inhibited the growth of LNCaP-32 by more than 50% (see Table 3). Earlier studies had shown that LNCaP cells were sensitized to undergo apoptosis by tumor necrosis factor α, TRAIL, and anti-Fas antibodies when Cx43 was expressed via adenoviruses to which Cx-null LNCaP cells were resistant [72]. Moreover, expression of Cx32 not only inhibits the growth of cells but also induces differentiation in breast cancer cell lines as well as in LNCaP cells [22], [73], [74].

What might be the possible explanation for the growth-suppressive effects of 1-25D with regard to the assembly of Cx32 into GJs? The signaling pathways that are activated or suppressed upon formation and degradation of GJs to impact cell growth and differentiation are not well-understood [16], [17], [75]–[79]. Elegant studies in Cx32 knockout mice revealed increased activation of mitogen-activated protein kinases and decreased level of tumor suppressor p27Kip1 [80]–[82]. While it is well-known that 1-25D suppresses the growth of several human PCA cell lines, the effect appear not to depend on the expression level of vitamin D receptor. For example, only LNCaP cells were found to be exquisitely sensitive to the growth inhibitory effect of 1-25D whereas other PCA cell lines, such as PC-3, DU-145 and ALVA-31, were barely sensitive despite the fact that all cell types expressed nearly similar levels of vitamin D receptor [50], [71], [83], [84]. Also, in LNCaP cells the growth suppression by 1-25D was mediated via increased expression of cyclin-dependent kinase inhibitors p21waf1/cip1 and p27kip1 as well as through hyper-phosphorylation of retinoblastoma protein, resulting in G0/G1 arrest [50]. Given the fact that Cx expression also has an impact on cell cycle [79], [85], it is possible that transmission of growth-regulatory signals through channels composed of Cx32 activates signaling pathways that increase the expression of gene-regulatory proteins involved in the control of cell cycle progression as proposed [18], [79]. Given the multiple effects of 1-25D on different tumor cell types, it is at present difficult to envisage how its chemopreventive, pro-differentiating and growth-inhibitory effects are related to its ability to regulate formation and degradation of GJs [16]–. More elaborate studies are underway to explore the molecular basis of the augmentation of growth-suppressive effect of 1-25D in LNCaP cells upon formation of GJs.

An intriguing observation made during this study was that 1-25D caused a radical change in the morphology of LNCaP-32 cells compared to Cx-null LNCaP-P and LNCaP-N cells such that the treated cells became flatter and acquired an epithelial morphology (Figure 9). Expression of Cx32 by itself had no conspicuous effect on the morphology and the change occurred only in response to 1-25D; and only when cells had been growing in 1-25D-containing medium for at least 4 days and have begun to be contact-inhibited and growth-arrested. One possible explanation for these data is that the formation of large GJs in response to 1-25D permits a more elaborate remodeling of the cortical actin network, which has emerged as a key regulator of cell morphology [87], [88]. This notion is supported by studies that utilized embryonic fibroblasts from Cx43 knockout mice in wound-healing studies. Fibroblasts from Cx43 knockout mice showed cell polarity defects as characterized by the failure of the microtubule organizing center to reorient with the direction of wound closure as well as failure of actin stress fibers to appropriately align at the wound edge [89]. Whether expression of Cx32 in LNCaP cells also governs cell shape in response to 1-25D through modulation of actin-cortex or microtubule network remains to be explored in future studies.

Several pre-clinical and clinical trials have suggested that a decrease in vitamin D_3_ levels contributes to the development and possibly to the progression of human PCA [1], [2], [90], [91]. Connexin32 is expressed by the luminal epithelial cells of normal prostate and is aberrantly assembled in the epithelial cells of prostate tumors [22], [23]. Because GJs have been implicated in maintaining the polarized and differentiated state of epithelial cells [39], we propose that the chemopreventive effects 1-25D in PCA may result from its ability to enhance the formation of GJs. Our results show that GJ assembly is the down-stream target of signaling initiated by 1-25D and that the formation of GJs sensitizes PCA cells to its growth modulatory influence. Because loss of cell junctions is a hallmark of PCA progression [29] and might occur as early as during prostatic intraepithelial neoplasia [92], [93], understanding basic cell and molecular biological mechanisms by which 1-25D might govern the formation of GJs will provide new insights with regard to signaling pathways utilized to maintain the polarized and the differentiated state of epithelial cells in prostate tumors. This should open innovative avenues for designing new therapeutic approaches to delay the onset of malignancy as loss of polarization is the earliest changes that may initiate PCA progression [92].
